# Adsorbing Volatile Organic Chemicals by Soluble Triazine-Based Dendrimers under Ambient Conditions with the Adsorption Capacity of Pyridine up to 946.2 mg/g

**DOI:** 10.3390/molecules26164862

**Published:** 2021-08-11

**Authors:** Yao-Chih Lu, Chia-Yun Chien, Hsiu-Fu Hsu, Long-Li Lai

**Affiliations:** 1 Department of Applied Chemistry, National Chi Nan University, No. 1 University Rd., Puli, Nantou 545, Taiwan; s103324901@mail1.ncnu.edu.tw (Y.-C.L.); s107324509@mail1.ncnu.edu.tw (C.-Y.C.); 2 Department of Chemistry, Tamkang University, No. 151, Yingzhuan Rd., New Taipei City 251, Taiwan; hhsu@mail.tku.edu.tw

**Keywords:** dendrimer, porous material, volatile organic chemical, pyridine

## Abstract

Two triazine-based dendrimers with peripheral 1,3,5-triamidobenzene (1-3-5-TAB) functionality were prepared, and their void spaces in the bulk solid were investigated. We examined dendrimers of three core lengths and determined the one with the longest core exhibits the largest void space because the peripheral amides were not imbedded in the internal space of each dendritic molecule. The new dendrimers as solids were observed to adsorb volatile organic chemicals efficiently. Importantly, because the dendrimers are soluble in organic solvents, the adsorbed VOCs can be quantified by ^1^H-NMR spectroscopy by choosing a chemical shift (δ) of dendrimers as the internal standard to exclude interfering impurity signals, a much simpler and more efficient protocol than the traditional GC technique for the VOC quantification. One dendrimer was found to adsorb 24 equivalents of pyridine, so its adsorption capacity is equivalent to 946.2 mg/g. This is a more than 2-fold increase than the reported values by other porous materials.

## 1. Introduction

Volatile organic chemicals (VOCs) are harmful to human health and the natural environment [1,2,3]. VOCs, including aromatics, alkanes, amines, and oxygenated or halogenated hydrocarbons [4,5,6], are often produced by chemical and pharmaceutical industries [7,8,9]. Porous materials currently used for adsorbing VOCs are zeolites (Zs) [10,11,12], activated carbons (ACs) [13,14,15,16], polymeric resins (PRs) [17,18,19,20], and metal-organic frameworks (MOFs) [21,22,23,24]. In these systems, a pre-drying column to remove the moisture of absorbed chemicals is generally required to maintain the adsorbing efficiency [24], which is an additional cost for VOC adsorption. For example, pyridine easily adsorbs moisture from its surroundings, which has been ascribed to the rarely reported works of pyridine-capturing by porous materials [20,24]. However, pyridine, widely used in the production of pharmaceuticals and pesticides, is known to cause serious neurological injury under chronic exposure [25]. Therefore, porous materials showing easy and efficient pyridine-adsorption are highly desired. On the other hand, dendrimers, generally with significant internal void spaces, are reported to adsorb metal ions or small molecules in solution for the purpose of catalyzing [26,27] and drug delivery [28,29,30]. As solids, dendrimers have also been demonstrated to contain dendritic void spaces and adsorb gases [31,32]. However, dendrimers have not yet been used as porous materials to adsorb VOCs. In particular, dendrimers with amide groups at their periphery may have good pyridine adsorbing ability [32]. Dendrimers constructed by covalent bonding between light atoms, such as carbon, oxygen, and nitrogen, showed low density and high thermal stability comparable to those of ACs and PRs. More importantly, dendrimers are generally soluble in organic solvents, and therefore are easy to be reprocessed and purified; these characteristics are not usually shown by Zs, ACs, PRs, and MOFs.

**Figure 1:**
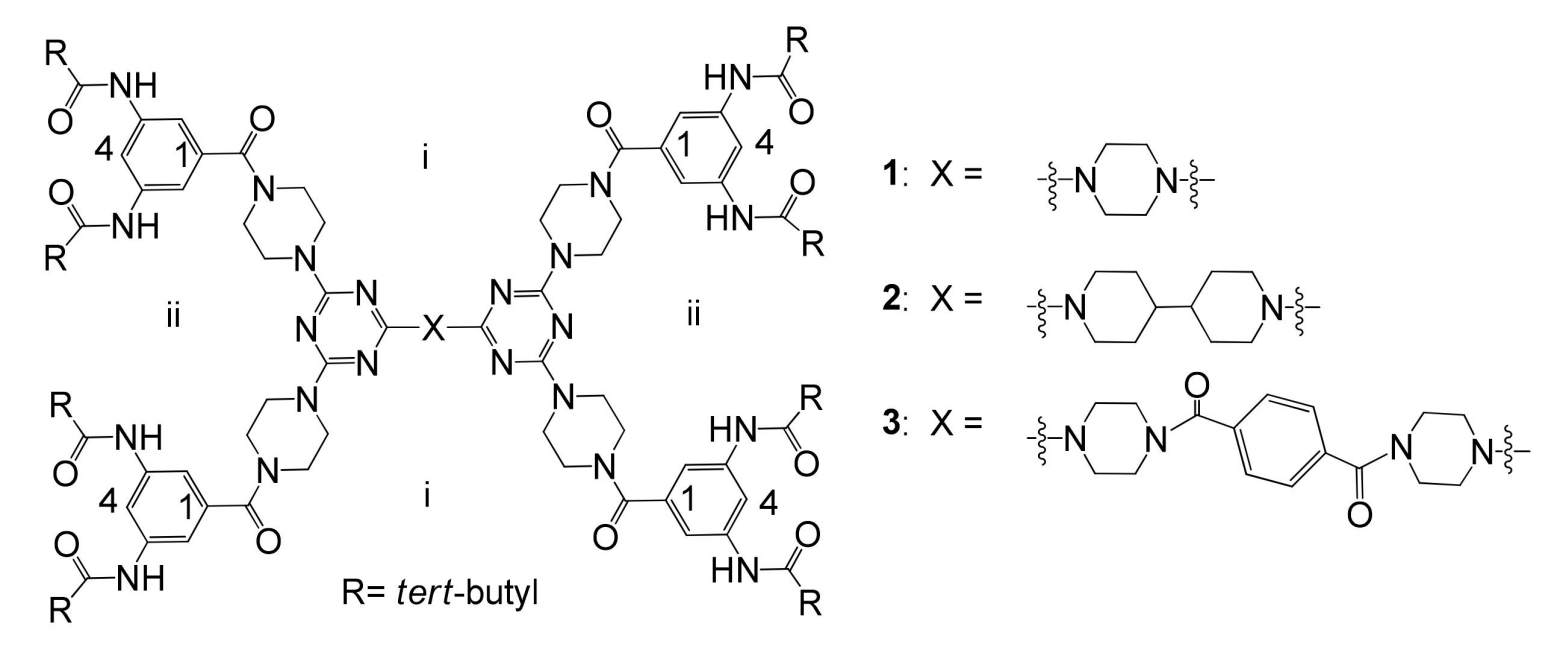
The molecular structures of dendrimers **1–3**.

Previously, we prepared dendrimer **1**, with piperazine in the central core and 1,3,5-triamidobenzene (1-3-5-TAB) moiety as a peripheral functionality (Figure 1) [32], showing the BET value of CO_2_ was coming mostly from the distribution of interstitial void space i of the molecules (Figure 1). The peripheral amides were not imbedded in the internal space of each dendritic molecule in the bulk solid, and the void space ii is much less accessible to gases [32]. In this report, to further increase the void spaces for adsorbing gases or VOCs, we have further designed and prepared dendrimer **2**, with 4,4′-biperidine in the central core and 1-3-5-TAB as the peripheral functionality. The corresponding i void space in dendrimer **2** is larger than that in dendrimer **1** and, as expected, **2** showed better BET values than **1**. However, **2** had a lower VOC adsorption capacity than **1**. To increase the VOC adsorption capacity, dendrimer **3**, with a longer chain length and polar functional groups in the central core, was prepared, and the corresponding VOC adsorption capacities were found to be better than those of dendrimers **1** and **2**. As the host dendrimers are soluble in organic solvents, ^1^H-NMR spectroscopic investigations were possible to quantify the VOC adsorptions. This is a much simpler and more efficient protocol than the traditional GC [21,22] and gravimetric gas analysis [24] techniques for VOC quantification. By properly choosing a chemical shift (δ) of dendrimers as the internal standard, the peak interferences from the H_2_O and impurity in the spectra can be easily avoided, and the accuracy of the adsorbed amount of VOCs can thus be ascertained. In particular, the adsorption capacity of pyridine for **3** is about 946.2 mg/g, a more than 2-fold increase from the reported values in the literature [20].

## 2. Experimental Section

### 2.1. General

Reagents were used as received without further purification. Elemental analyses were performed using an Unicube analyzer EA000600 (Elementar, Langenselbold, Germany). The spectra of ^1^H and ^13^C NMR were recorded on a AMX-300 spectrometer of National Chi Nan University (Bruker, Billerica, MA, USA). Thermogravimetric analyses were completed under N_2_ with a TGA-7 TG analyzer (Perkin Elmer, Waltham, MA, USA). The mass spectra were obtained from Microflex MALDI-TOF MS (Bruker, Billerica, MA, USA). The FT-IR spectra were recorded by a Perkin Elmer Frontier spectrometer (Perkin Elmer, Waltham, MA, USA). Brunauer-Emmett-Teller (BET) analyses were performed by a TriStar II Plus system (Micromeritics Instrument Corporation, Norcross, GA, USA) using CO_2_ as the adsorbate at 195, 273, and 298 K, respectively, and N_2_ as the adsorbate at 77 K. All gases for experiments were pure up to 99.9995%.

### 2.2. Preparation of Dendrimer **2**

4,4′-Bipiperidine (0.085 g, 0.5 mmol) was dissolved in THF (50 mL) and added to dendron **4** (0.890 g, 1.0 mmol) and K_2_CO_3_ (0.380 g, 2.5 mmol) in THF (50 mL), and the resulting mixture was heated at 70 °C for 48 h. After reaction, solvent was removed at reduced pressure, and water (50 mL) was then added. The mixture was added to water to produce a solid, which was filtered off and then washed with THF (5 mL × 2). Dendrimer **2** thus obtained a 70.2% yield (0.657 g). ^1^H-NMR (300 MHz, CDCl_3_, 25 °C, TMS): δ = 8.00 (s, 4H, 4 × Ar-H), 7.94 (br. s, 8H, 8 × NH), 7.41 (s, 8H, 8 × Ar-H), 4.68–4.64 (m, 4H, 2 × CH_2_), 3.73–3.48 (m, 34H, 16 × CH_2_, 2 × CH), 2.65–2.62 (m, 4H, 2 × CH_2_), 1.70 (br. s, 4H, 2 × CH_2_), 1.27 (s, 72H, 24 × CH_3_), 1.12 (br. s, 4H, 2 × CH_2_) ppm; ^13^C-NMR (75 MHz, CDCl_3_, 25 °C, TMS): δ = 177.49, 169.99, 165.46, 165.02, 139.28, 136.67, 114.06, 112.55, 48.03, 43.77, 43.04, 42.43, 19.90, 29.42, 27.66 ppm; FT-IR: 3453 and 3328 cm^−1^ (s. N-H), 3078 cm^−1^ (w. Ar-H), 2968, 2935, 2910 and 2871 cm^−1^ (m. C-H), 1662, 1630 and 1610 cm^−1^ (s. C=O), 1590 cm^−1^ (s. C=N); MS: *m/z*: calcd for C_100_H_142_N_24_O_12_ (M)^+^: 1871.4; found: 1870.9; elemental analysis: calcd (%) for (C_100_H_142_N_24_O_12_ + 6H_2_O) C 60.65, H 7.84, N 16.97; found: C 60.51, H 7.78, N 17.00; m.p.: 317.6–319.5 °C.

### 2.3. Preparation of Dendrimer **3**

Terephaloyl chloride (0.052 g, 0.25 mmol) in dried THF (25 mL) was added to dendron **5** (0.493 g, 0.5 mmol) in dried THF (25 mL), then stirred in an ice-bath for 30 min. Triethylamine (0.210 mL, 1.5 mmol) was added, and the resulting solution was stirred at room temperature for another 2 h. Solvent was then removed at reduced pressure, and water (50 mL) was then added to yield a solid, which was further purified by chromatography (column 10 × 2.1 cm; eluent, THF-CH_2_Cl_2_: 1:2). Dendrimer **3** obtained a 47.2% yield (0.474 g). ^1^H-NMR (300 MHz, CDCl_3_, 25 °C, TMS): δ = 7.99 (s, 4H, 4 × Ar-H), 7.77 (br. s, 8H, 8 × NH), 7.47–7.45 (m, 12H, 12 × Ar-H), 3.74–3.40 (m, 48H, 24 × CH_2_), 1.28 (s, 72H, 24 × CH_3_) ppm; ^13^C-NMR (75 MHz, CDCl_3_, 25 °C, TMS): δ = 177.43, 169.91, 165.25, 139.23, 136.62, 127.50, 114.26, 112.60, 47.94, 43.66, 42.40, 39.90, 27.66 ppm; FT-IR: 3444 and 3320 cm^−1^ (s. N-H), 3080 cm^−1^ (w. Ar-H), 2968, 2933, 2909 and 2871 cm^−1^ (m. C-H), 1666, 1636 and 1611 cm^−1^ (s. C=O), 1600 cm^−1^ (s. C=N); MS: *m/z*: calcd for C_106_H_144_N_24_O_14_ (M)^+^: 2005.5; found: 2006.4; elemental analysis: calcd (%) for (C_106_H_144_N_26_O_14_ + 6H_2_O) C 60.21, H 7.44, N 17.22; found: C 60.21, H 7.52, N 17.23; m.p.: 369.8–371.5 °C.

## 3. Results and Discussion

**Scheme 1:**
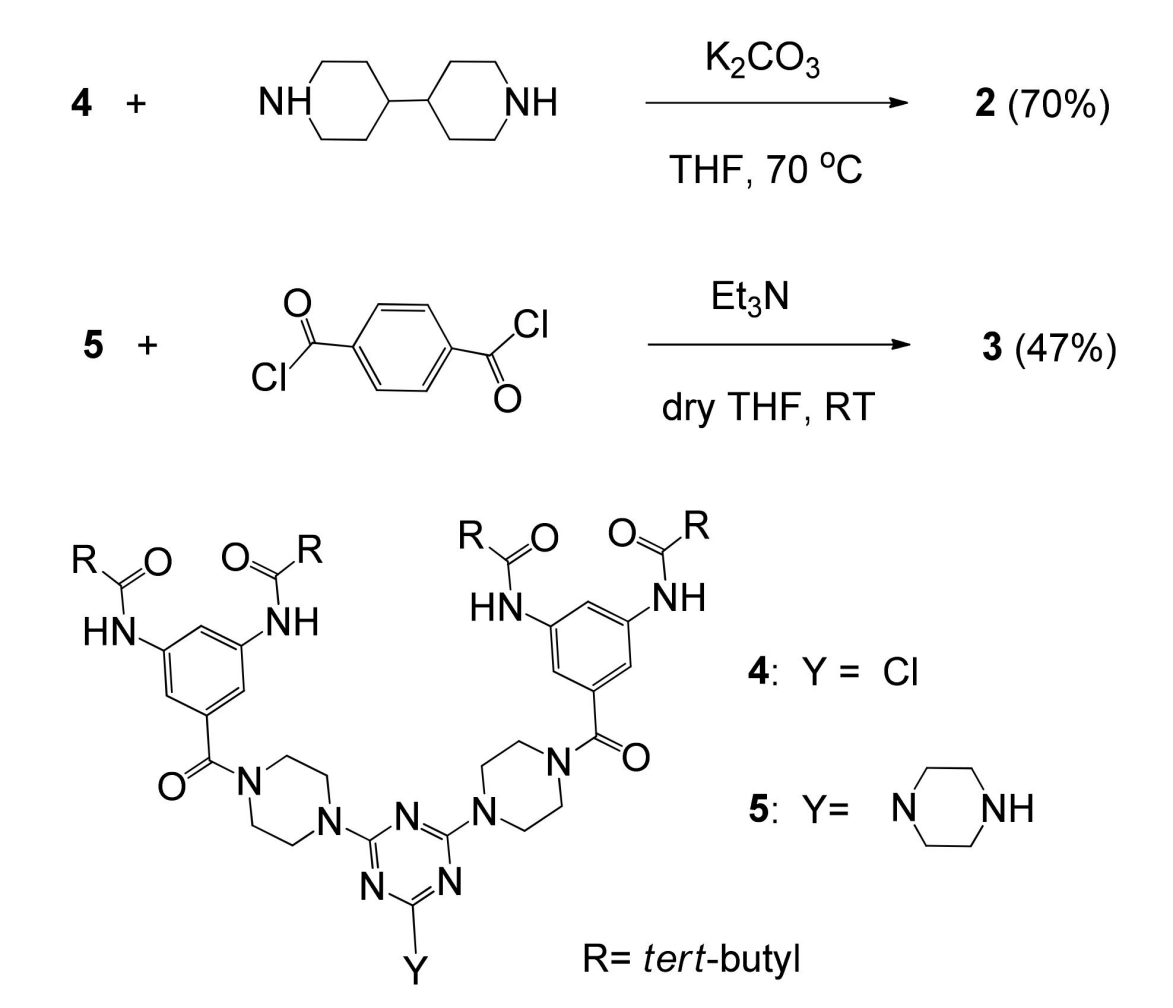
Preparation of dendrimers **2** and **3**.

Dendrons **4** and **5** were prepared according to the literature [32]. Two equivalents of **4** reacted with 4,4′-bipiperidine in the presence of potassium carbonate in THF to lead the formation of dendrimer **2** in 70% yield. Treatment of two equivalents of **5** with terephaloyl chloride in the presence of triethylamine in dried THF produced dendrimer **3** in 47% yield (Scheme 1). Dendrimers **2** and **3** were characterized by ^1^H- and ^13^C-NMR spectroscopies, elemental analysis, and mass spectrometry.

**Figure 2:**
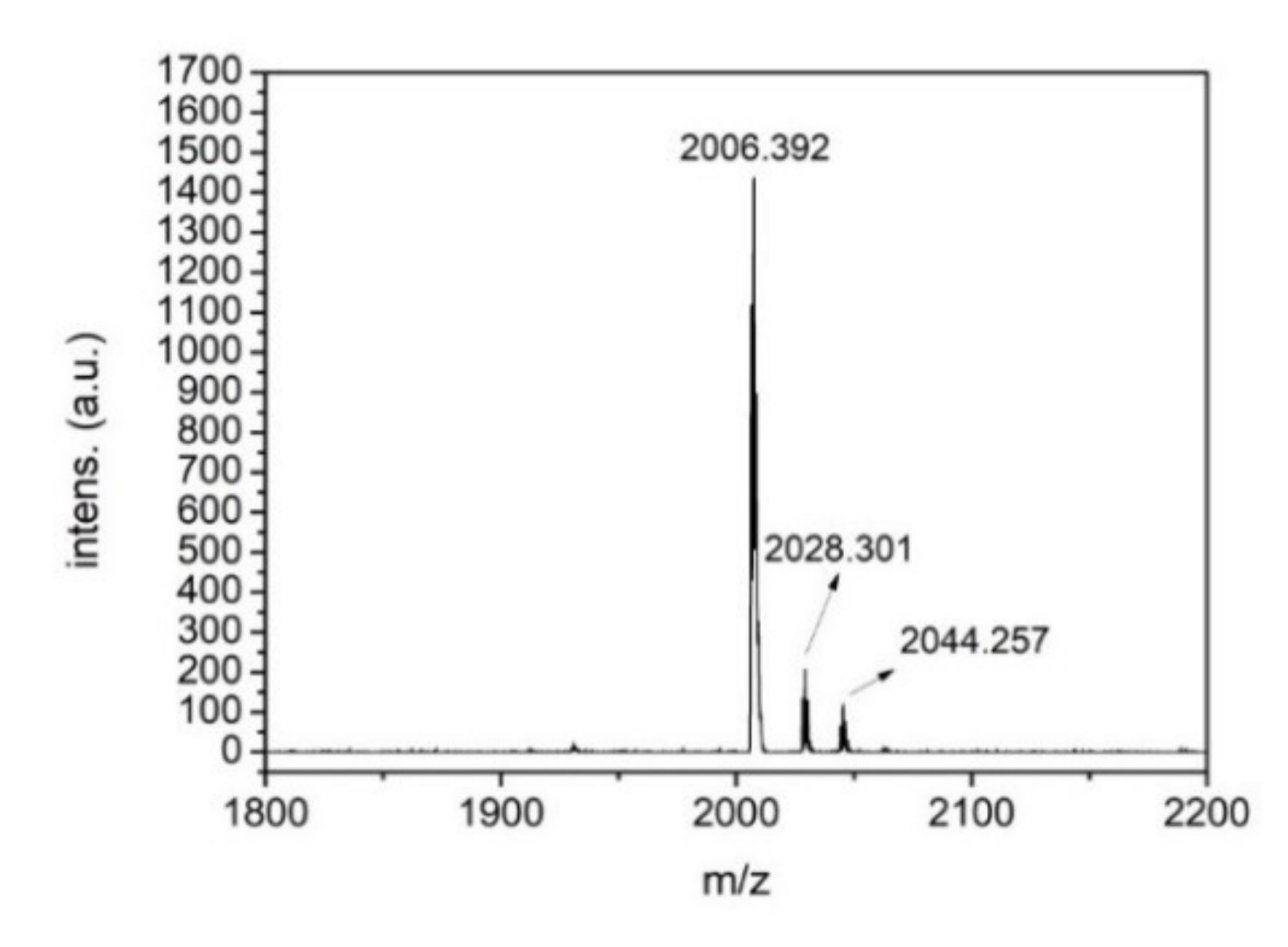
The mass spectrum of dendrimer **3**.

**Figure 3:**
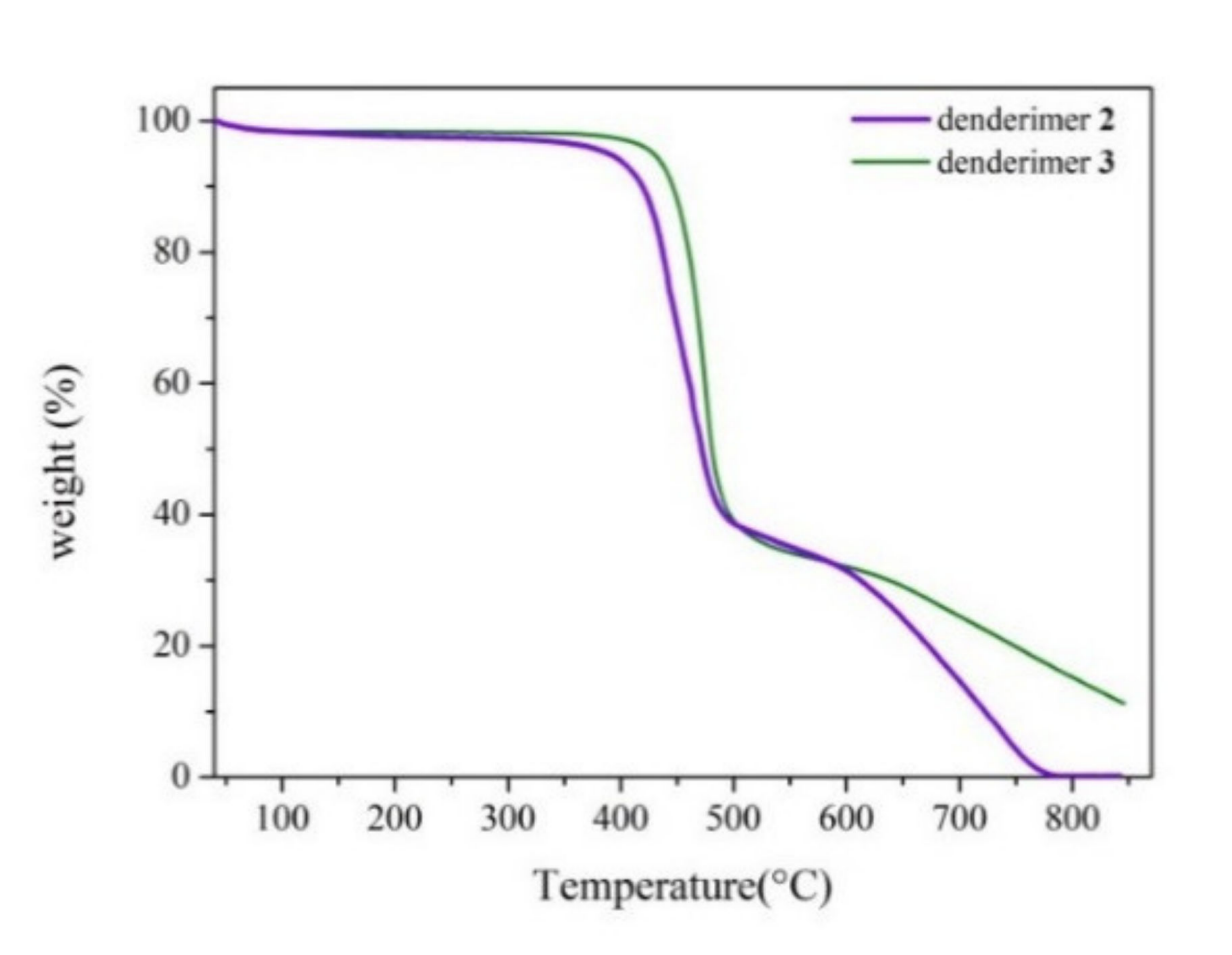
Thermogravimetric analysis of dendrimers **2** and **3** from 30 to 850 °C at a heating rate of 10 °C min^−1^ under an N_2_ atmosphere.

The mass spectrum of **3** in Figure 2, obtained by MALDI-TOF as an example, clearly shows three peaks of *m/z* at 2006.4, 2028.3, and 2044.3 from [M]^+^, [M+Na^−^H]^+^, and [M+K^−^H]^+^ ions, respectively. Dendrimers **2** and **3** are stable up to 300 °C, as evidenced by TGA analysis (Figure 3), and the temperatures showing 5% decomposition of dendrimers **2** and **3** are about 370 and 430 °C, respectively. The slight weight loss below 120 °C probably comes from the vaporization of water because amide groups at their periphery adsorb moisture easily, as further confirmed by elemental analysis. The error percentages of C, H, and N between experimental and theoretical values of **2**·6H_2_O or **3**·6H_2_O are all within 0.2%. Interestingly, the residue of TGA from dendrimer **3** was at about 15 wt% at 800 °C, but the residue from dendrimer **2** dropped to zero at about 770 °C. The central core of **2**, bipiperidine, is probably more flexible and therefore more accessible to be burned off completely.

**Figure 4:**
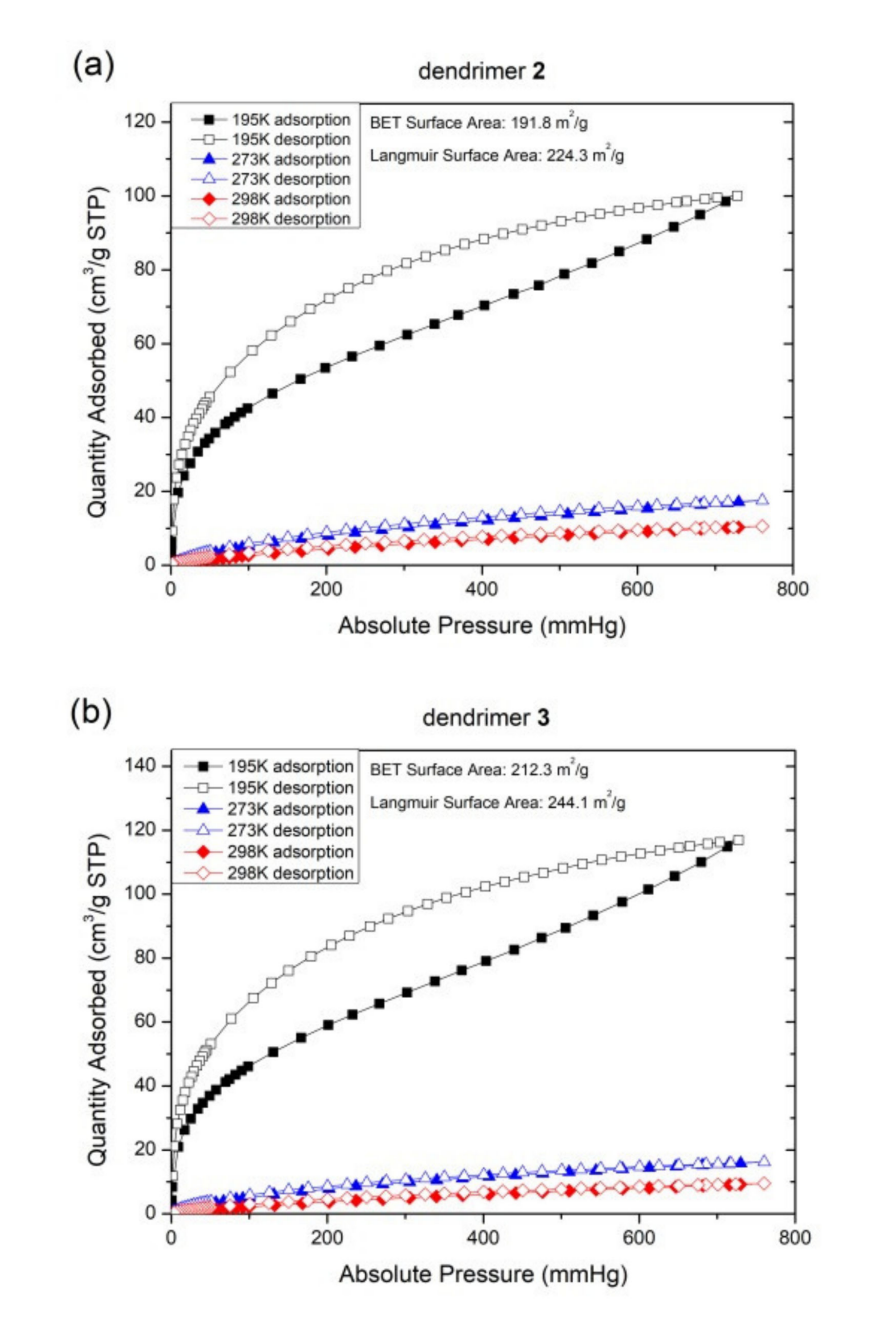
(**a**) The CO_2_ sorption isotherms of dendrimer **2** at 195, 273, and 298 K. (**b**) The CO_2_ sorption isotherms of dendrimer **3** at 195, 273, and 298 K.

Dendrimers **2** and **3** were degassed at 110 °C under a vacuum for 3 h, and their CO_2_ gas adsorption behaviors were then studied (Figure 4). The Brunauer-Emmett-Teller (BET) surface area of **2** on the basis of its CO_2_ adsorption at 195 K was calculated to be 191.8 m^2^/g using the method in the literature [33,34,35]. In the same manner, the BET surface area of **3** was estimated to be 212.3 m^2^/g. The BET surface areas of dendrimers **2** and **3** were 41 and 56% higher, respectively, than that of **1** (136.0 m^2^/g) [32].

**Figure 5:**
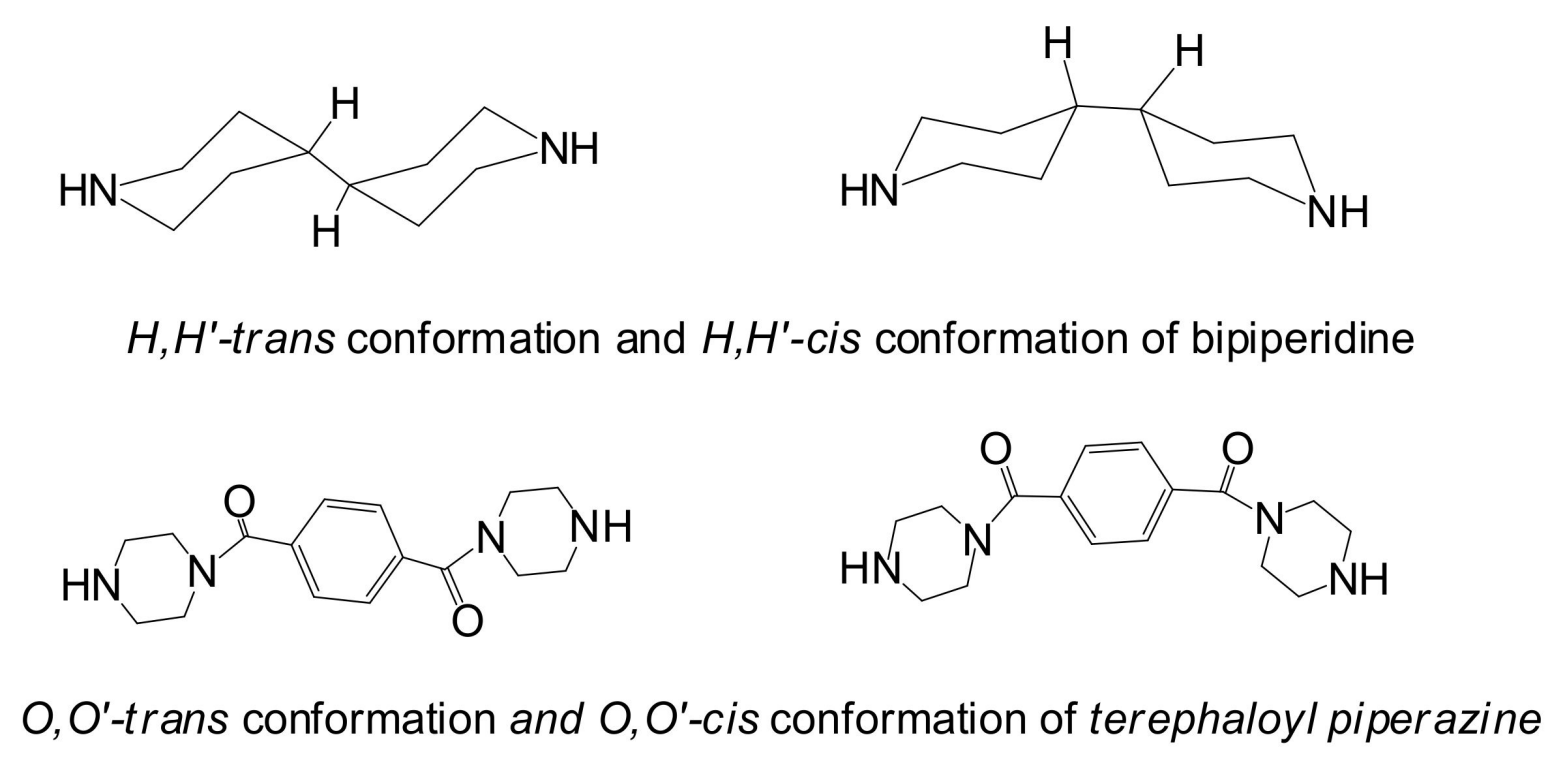
The *cis* and *trans* of 4,4′-bipiperidine and terephaloyl piperazine.

**Figure 6:**
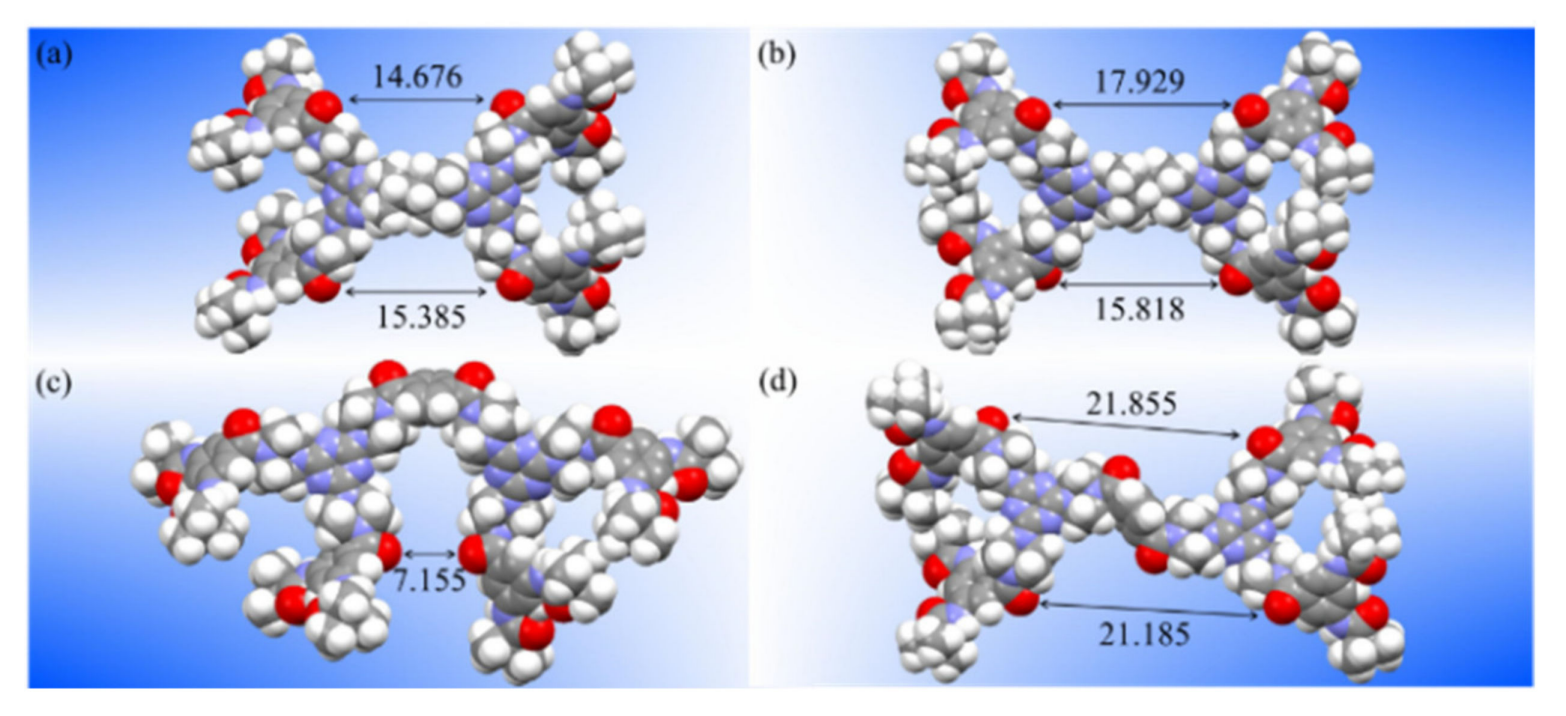
The molecular conformations of dendrimers **2** and **3** in a space-filled model, O: red, C: gray, N: purple, H: white; (**a**) the *H,H′-cis* of **2**; (**b**) the *H,H′-trans* of **2**; (**c**) the *O,O′-cis* of **3**; (**d**) the *O,O′-trans* of **3**.

The conformations of dendrimers **2** and **3** were studied to further elucidate the effect of central cores on void spaces of dendritic molecules. As demonstrated in the optimization of dendrimer **1** by CaChe using the MM2 model [32], the conformation of **2** was obtained by bonding two pre-optimized dendrons, **4** and 4,4′-bipiperidine, followed by optimization. Since 4,4′-bipiperidine can be in two possible *H,H′-cis* and *H,H′-trans* isomeric forms (Figure 5), two corresponding conformations of dendrimer **2** were optimized. The heat of formation for **2** with the *H,H′-cis* isomer is −22.11 Kcal/mol and that for the *H,H′-trans* isomer is −26.71 Kcal/mol. Analogously, terephaloyl piperazine can be in two possible isomeric forms, i.e., *O,O′-cis* and *O,O′-trans* conformations (Figure 5), so two isomers of dendrimer **3** were optimized. The heat of formation for the *O,O′-cis* isomer of **3** is 1.45 kcal/mol, and that for the *O,O′-trans* isomer is −2.10 kcal/mol (Figure 6). Since the *trans* isomer of both dendrimers is more stable than the corresponding *cis* isomer, dendrimers **2** and **3** exist predominantly as the *trans* forms in their bulk solids. Although simulations in CaChe were carried out in the gas phase and the conformations of dendrimers in bulk solids may differ from the optimized conformations, the results can be used to qualitatively correlate the relative BET values of dendrimers **2** and **3**. As mentioned in the literature [32], several strong H-bond interactions for constructing the porous pores of dendrimer **1** arise between the C=O and NH moieties of the 1-3-5-TAB moiety. Therefore, the space around the 1-3-5-TAB unit may not be available for gas access in dendrimers **2** and **3**, and the distances between two oxygen atoms of two C=O groups, to some extent, explain the void space in one dimension of both dendrimers in the solid state. Two O…O distances in the *H,H′-trans* conformation of **2** are about 17.9 and 15.8 Å, respectively, and those of the *O,O′-trans* conformation of **3** are about 21.9 and 21.2 Å, respectively (Figure 6). The O…O distances in the *O,O′-trans* conformation of **1** are about 12.2 and 13.0 Å (Figure S1) [32]. Based on the optimized conformations, the void space in the bulk solid of dendrimers **1**–**3** is in the order of **3** > **2** > **1**, consistent with the BET measurements.

To study the VOC adsorbing behaviors of dendrimers, dendrimer **1** as a model compound was recrystallized from hexane-THF (19:1), vacuumed at 110 °C for 3 h, then ^1^H-NMR spectroscopy confirmed the complete removal of solvents. To adsorb the vapor of VOC, the vacuumed **1** (≈6.5 mg) was then maintained at room temperature (≈28 °C) in a closed bottle (10 mL) containing a small amount of VOC (≈1 mL) in an isolated bottle (2 mL). After 18 h, the resulting dendrimer was dried in a fume hood for 10 min and then dissolved in CDCl_3_ or DMSO-D_6_ for ^1^H-NMR spectroscopy measurement. A specific chemical shift (δ) of the dendrimer, away from and therefore excluding the signal interferences from H_2_O and impurities, was used as an internal standard to quantify the amount of adsorbed VOC. For example, the intensity of the chemical shift at ≈8.1 ppm from the H (at C_4_) between two amide substituents of dendrimer **1** (Figure 1) was used to compare with the intensity of *o*-H (≈8.2 ppm) of nitrobenzene, and the quantification of adsorbed nitrobenzene could then be obtained. As these two chemical shifts from dendrimer **1** and nitrobenzene, respectively, are away from those of H_2_O and other impure hydrocarbons, the interferences from H_2_O and impurities can be excluded. Accordingly, other adsorbed VOCs by dendrimers **1**–**3** could be thus quantified. As shown in Table 1, one molecule of dendrimer **1** can adsorb 4, 11, 3 and 0.6 equivalents of nitrobenzene, pyridine, toluene, and hexane, respectively (Figure S2). In a similar manner, one molecule of dendrimer **2** was found to adsorb 3, 7, 2, and 0.3 equivalents of the corresponding VOCs. Based on the BET study and simulation, the void space of dendrimer **2** in the bulk solid is larger than that of dendrimer **1**. However, the adsorption capacities of **2** for nitrobenzene, pyridine, toluene, and hexane are slightly lower than those of **1**. It has been reported that adsorption capacities of porous materials could depend on their void spaces and the functional groups on the surface of the porous substrate [36,37]. To achieve higher adsorption capacity, dendrimer **3** with terephaloyl piperazine in the central core was prepared. One molecule of **3** was found to adsorb 4, 24, 5, and 0.8 equivalents of nitrobenzene, pyridine, toluene, and hexane, respectively (Figure S2). The amide functionality of terephaloyl piperazine not only enlarges the void space of bulk dendrimers but also enhances the interaction between VOCs and the porous substrate. The adsorption capacity of **3** in pyridine is equivalent to 946.2 mg/g, a more than 2-fold increase from the reported value in the literature (400.8 mg/g) [20]. Although dendrimer **3** has a significantly smaller surface area than MOFs [36,37,38,39,40], its void space may increase in the process of adsorbing VOCs because its void pore is formed by flexible H-bonding interactions between dendritic peripheral groups, as evidenced by the liquid formation and pyridine adsorption onto bulk solid **3**. The adsorption capacities of **3** in nitrobenzene and toluene were also calculated to be 273.4 and 229.6 mg/g, respectively, based on ^1^H-NMR spectroscopy, which are less than the reported values of nitrobenzene (344 mg/g)^13^ and toluene (1096 mg/g)^38^. It is worthwhile to note that no significant decompositions of dendrimers **1**-**3** after adsorbing VOCs were observed, as demonstrated by the related ^1^H-NMR spectra (Figure S2). Therefore, VOC-containing dendrimers are expected to be recovered for further VOC adsorption

**Figure 7:**
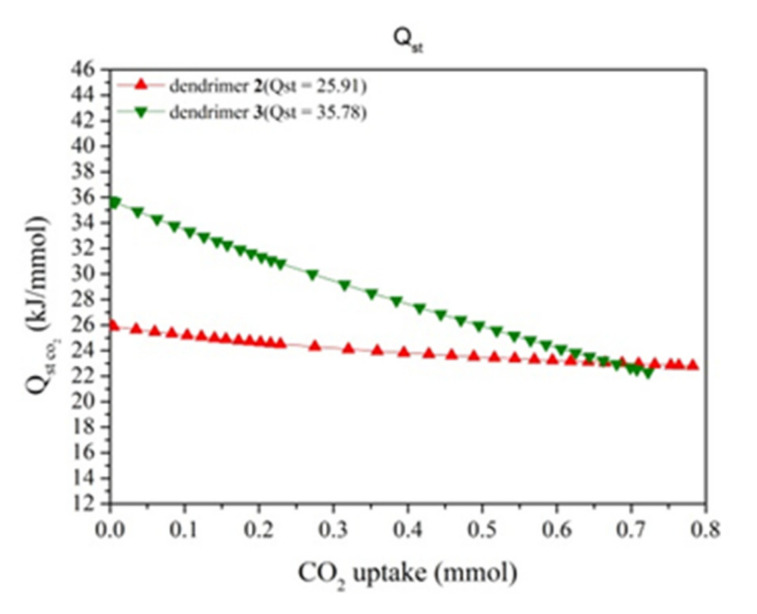
The isosteric heat (*Q*_st_) of CO_2_ sorption of dendrimers **2** and **3**.

To further understand why dendrimers **2** and **3** have different adsorbing ability in pyridine, the isosteric heats of CO_2_ sorption (*Q*_st_) were calculated by the virial method (Scheme S1) [41,42,43,44]. Based on the CO_2_ adsorptions at 273 K and 298 K (Figure 4), the *Q*_st_ of **3** was measured to be 35.8 kJ/mol at zero coverage, and the *Q*_st_ of **2** was only 25.9 kJ/mol at zero coverage (Figure 7). Both *Q*_st_ values decreased when more CO_2_ gas was adsorbed by dendrimers **2** and **3**. At about 0.7 mmole uptake, the *Q*_st_ of **3** is similar to that of **2**, about 24.0 kJ/mol for both dendrimers, which agrees with our previous result that the void spaces from i in their bulky stackings are more easily accessed by CO_2_ gas than those from ii [32]. In i void spaces, compared to **2**, dendrimer **3** possesses two additional amide groups in the dendritic core to enhance the interaction with CO_2_ molecules in the presence of triazine moieties. Therefore, the *Q*_st_ of **3** is higher than that of **2** below 0.6 mmole uptake. When most void spaces from i are filled up, a small amount of CO_2_ molecules may go into the void spaces from ii, and at this stage, the *Q*_st_s of **3** and **2** are similar.

According to our previous study [32], the *Q*_st_ of **1** was 27.8 kJ/mol at zero coverage, which is slightly larger than that of **2** but much smaller than that of **3**. This trend is in agreement with the adsorption capacity of dendrimers **1**–**3**. Two additional amide groups in the central core of dendrimer **3** also strengthen the interaction of pyridine in the presence of the triazine moiety, greatly increasing the adsorption capacity of pyridine. However, the adsorption capacities of dendrimer **3** in nitrobenzene, toluene, and hexane are much less. The crystallographic analysis of **3** may provide useful information for the difference. However, an attempt to grow crystals from **3** for a single crystal structure determination was not successful, and only powders from **3** were obtained. The N_2_ sorption isotherms of dendrimers **2** and **3** at 77 K show that there is almost no N_2_ adsorption for **2** (Figure S3). Therefore, the pore size distribution is estimated by the adsorption of nitrogen by dendrimer **3**. There are two major pore sizes for **3**: the first is ≈5.8 Å and the second is ≈7.5 Å (Figure S4). The peak intensity of the first is greater than that of the second. In addition, its distribution is quite broad, indicating the porous framework is quite flexible and may change under VOC absorption, as previously mentioned. The molecular sizes of nitrobenzene and toluene are slightly larger than that of pyridine, so the adsorption capacities of nitrobenzene and toluene are less than that of pyridine. Hexane does not provide any strong interaction functionality with the amide and triazine moiety of the porous substrate, and thus its adsorption capacity can be almost negligible.

## 4. Conclusions

In summary, we have successfully prepared two new triazine-based dendrimers. The one with a longer core length possesses the amide functionality in the central core. It therefore has greater void space, more N-containing substrates, and better adsorption capacity of pyridine in the bulk solid. In particular, the adsorption capacity of pyridine is equivalent to 946.2 mg/g, which is much better than the reported values in the literature. The characteristics of easy preparation, purification, and reprocessing in dendrimer **3** make it a potential candidate for applications in sensing or sequestrating pyridine from VOCs. The void pores of bulk dendrimers are constructed by flexible intermolecular H-bond or π–π interactions and therefore able to be potentially enlarged in the course of adsorbing VOCs, as demonstrated in the present case. In addition to zeolites, activated carbons, polymeric resins, and metal-organic frameworks, porous dendrimers are promising for further studies in the adsorption of VOCs. Because dendrimers are generally soluble in organic solvents, their adsorption of VOCs can be easily quantified by ^1^H-NMR spectroscopy. It was also discovered that the isosteric heats (*Q*_st_) of the dendrimer based on the CO_2_ sorption may provide criteria for exploiting potential materials for VOC adsorption because the *Q*_st_ of porous materials is proportional to the adsorption capacity of VOCs in our triazine-based system.

## Figures and Tables

**Table 1:** The VOC adsorption of dendrimers **1**–**3**.

	Guest	Nitrobenzene	Pyridine	Toluene	Hexane
Host					
**1** in DMSO-D_6_		4	11	3	0.6
**2** in DMSO-D_6_		3	7	2	0.3
**3** in CDCl_3_		4	24	5	0.8

## Data Availability

The data presented in this study are available on request from the corresponding author.

## Supplementary Materials

The following are available online, Figure S1: The molecular conformations of dendrimer **1** in space-filled model, Figure S2: The ^1^H-NMR spectra of dendrimers **1–3** after adsorbing VOCs, Figure S3: The N_2_ sorption isotherms of dendrimers **2** and **3** at 77K, Figure S4: The pore size distribution of dendrimer **3** under nitrogen, Figure S5: The ^1^H-NMR and ^13^C-NMR spectra of dendrimers **2** and **3**, Scheme S1: Estimation of isosteric heats of gas adsorption.

## Sample Availability

Samples of the compounds **2** and **3** are available from the authors.
