# Supplementary material for: Adsorbing Volatile Organic Chemicals by Soluble Triazine-Based Dendrimers under Ambient Conditions with the Adsorption Capacity of Pyridine up to 946.2 mg/g
Source: Molecules. 2021 Aug 11;26(16):4862. doi: 10.3390/molecules26164862 (PMC8398626; doi:10.3390/molecules26164862)
Supplement: Supplementary file 1 [file molecules-26-04862-s001.zip › molecules-1327073-supplementary.pdf]

molecules

Support Information

Adsorbing volatile organic chemicals by soluble triazine-based dendrimers under ambient conditions with the adsorption capacity of pyridine up to 946.2 mg/g

Yao-Chih Lu, Chia-Yun Chien, and Long-Li Lai*

Department of Applied Chemistry, National Chi Nan University, No. 1 University Rd., Puli, Nantou 545, Taiwan; s103324901@mail1.ncnu.edu.tw (Y. L.); s107324509@mail1.ncnu.edu.tw (C. C.)
* Correspondence: lilai@ncnu.edu.tw (L. L.); Tel.: +886-49-2910960#4976

Figure S1. The molecular conformations of dendrimer **1** in space-filled model.......................... 2

Figure S2. The ^{1}H-NMR spectra of dendrimers **1-3** after adsorbing VOCs. 2

Scheme S1. Estimation of isosteric heats of gas adsorption. 14

Figure S3. The N_2 sorption isotherms of dendrimers **2** and **3** at 77K. 15

Figure S4. The pore size distribution of dendrimer **3** under nitrogen. 15

Figure S5. The ^{1}H-NMR and ^{13}C-NMR spectra of dendrimers **2** and **3.** 16

Figure S1. The molecular conformations of dendrimer **1** in space-filled model, O:red, C: gray, N: purple, H: white.

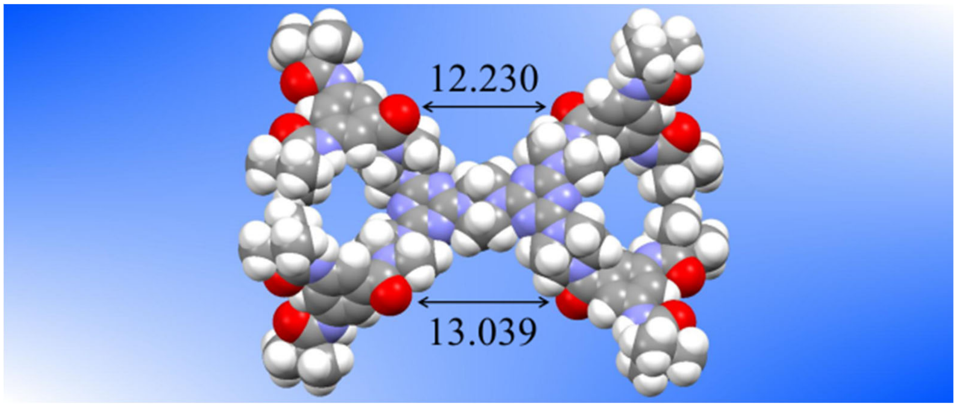

Figure S2. The ^{1}H-NMR spectra of dendrimers **1-3** after adsorbing VOCs.

(A) Adsorbing Nitrobenzene:

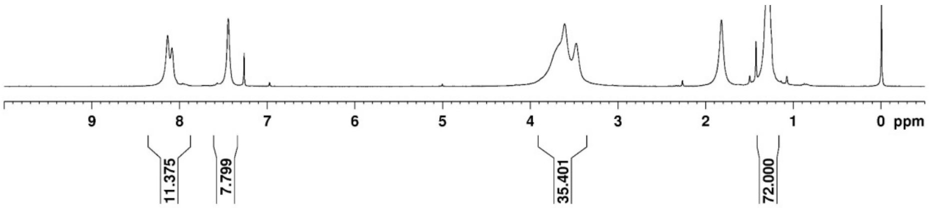

^{1}H-NMR of dendrimer **1** $CDCl_3$

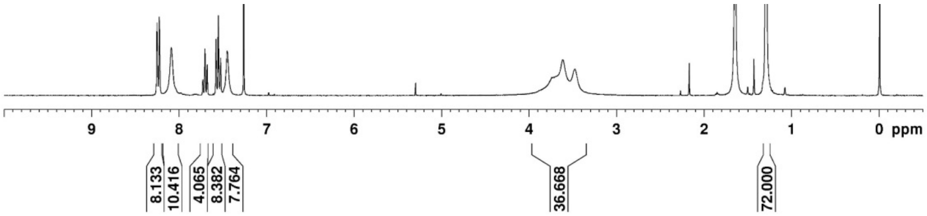

^{1}H-NMR of dendrimer **1** after adsorbing nitrobenzene in $CDCl_3$

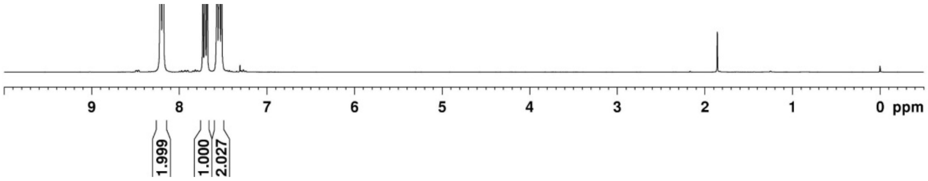

^{1}H-NMR of nitrobenzene in $CDCl_3$

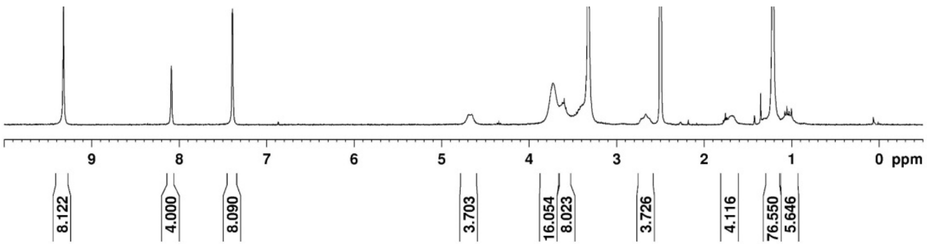

^{1}H-NMR of dendrimer **2** in DMSO-D_6

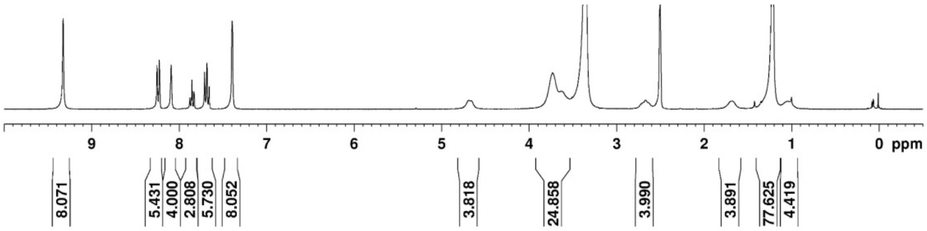

^{1}H-NMR of dendrimer **2** after adsorbing nitrobenzene in DMSO-D_6

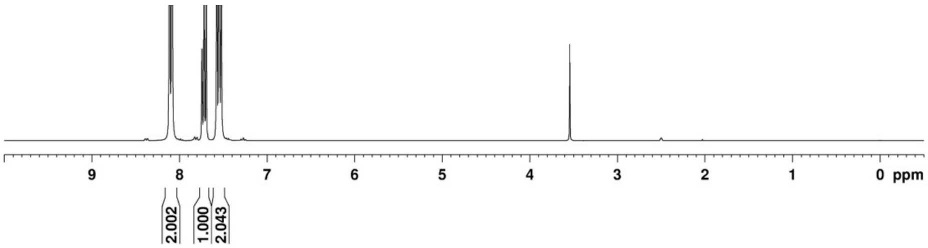

^{1}H-NMR of nitrobenzene in DMSO-D_6

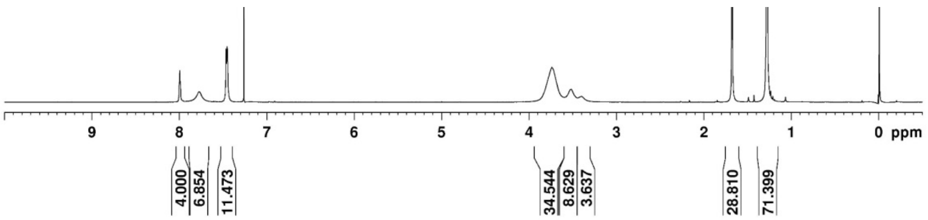

^{1}H-NMR of dendrimer **3** in $CDCl_3$

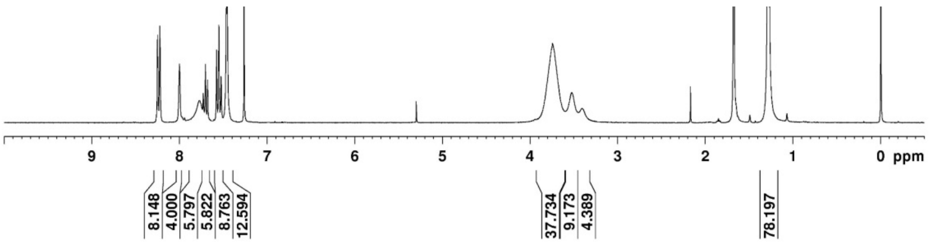

^{1}H-NMR of dendrimer **3** after adsorbing nitrobenzene in $CDCl_3$

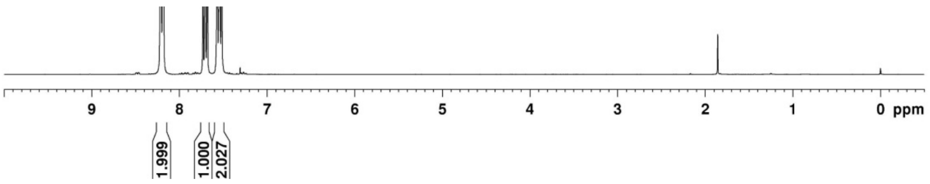

^{1}H-NMR of nitrobenzene in $CDCl_3$

(B) Adsorbing pyridine:

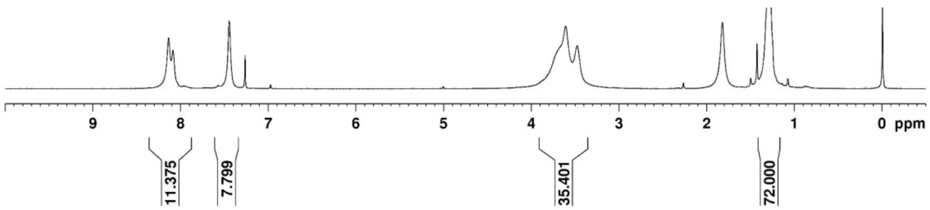

^{1}H-NMR of dendrimer **1** in $CDCl_3$

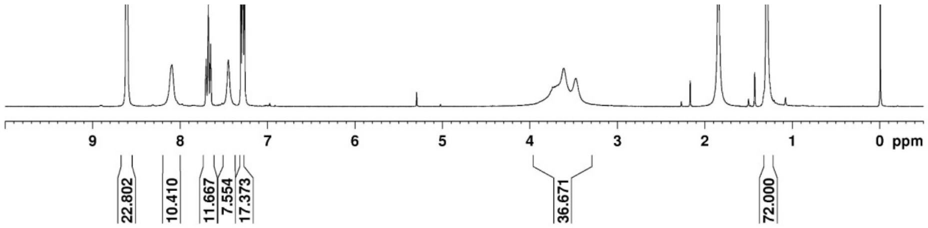

^{1}H-NMR of dendrimer **1** after adsorbing pyridine in $CDCl_3$

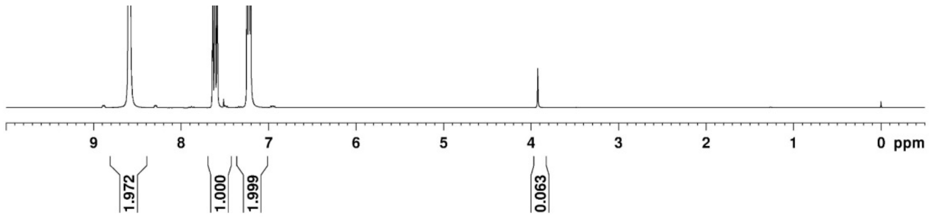

^{1}H-NMR of pyridine in $CDCl_3$

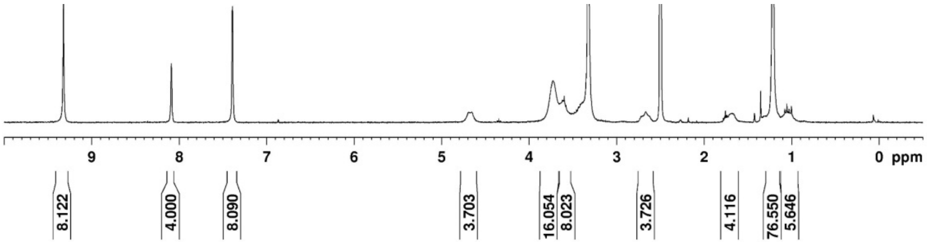

^{1}H-NMR of dendrimer **2** in DMSO-D$_6$

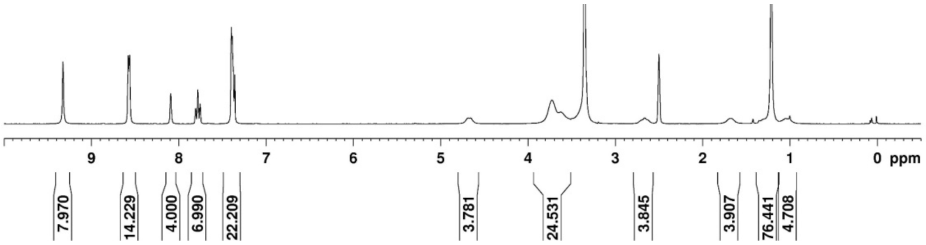

^{1}H-NMR of dendrimer **2** after adsorbing pyridne in DMSO-D$_6$

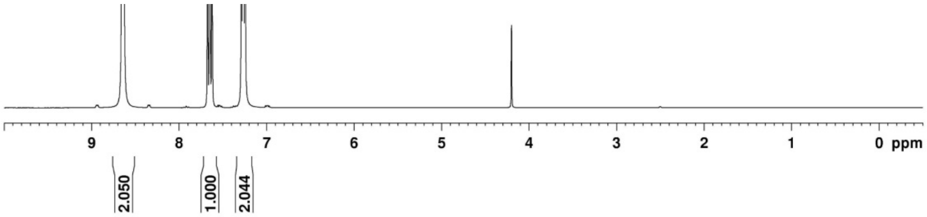

^{1}H-NMR of pyridne in DMSO-D$_6$

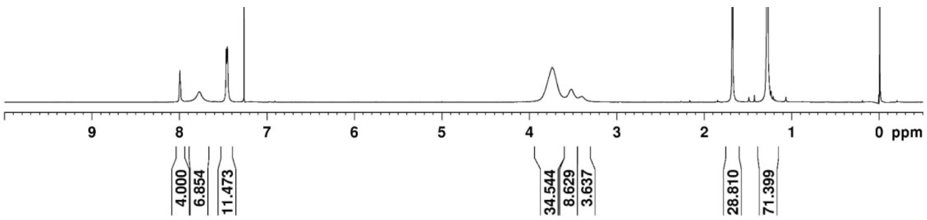

^{1}H-NMR of dendrimer **3** in $CDCl_3$

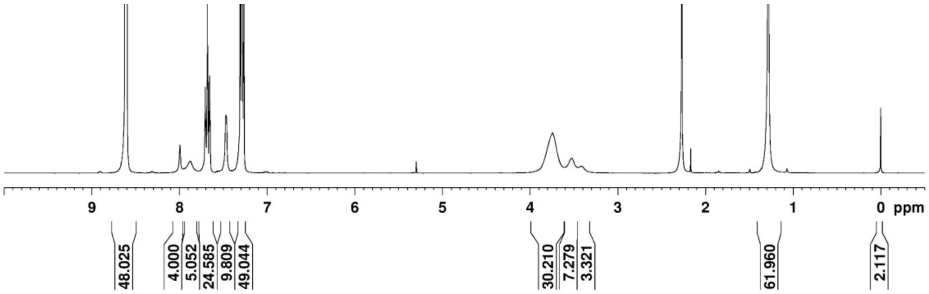

^{1}H-NMR of dendrimer **3** after adsorbing pyridne $CDCl_3$

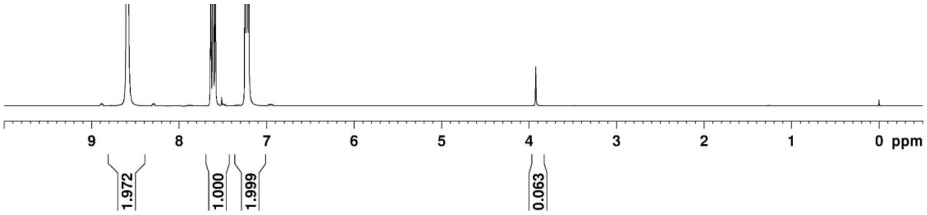

^{1}H-NMR of pyridine $CDCl_3$

(C) Adsorbing Toluene:

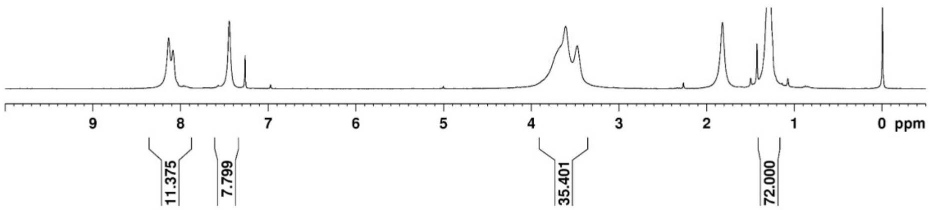

^{1}H-NMR of dendrimer **1** $CDCl_3$

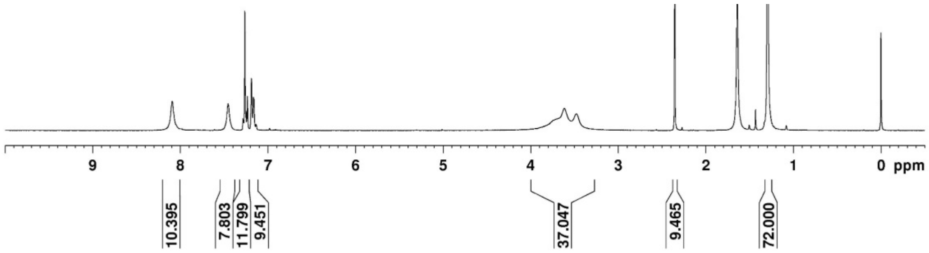

^{1}H-NMR of dendrimer **1** after adsorbing toluene in $CDCl_3$

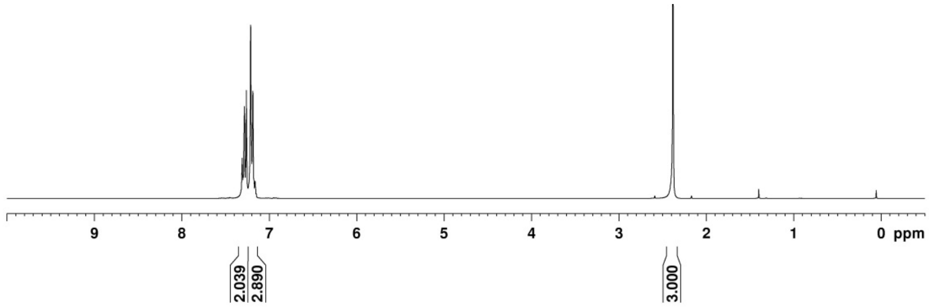

^{1}H-NMR of toluene in $CDCl_3$

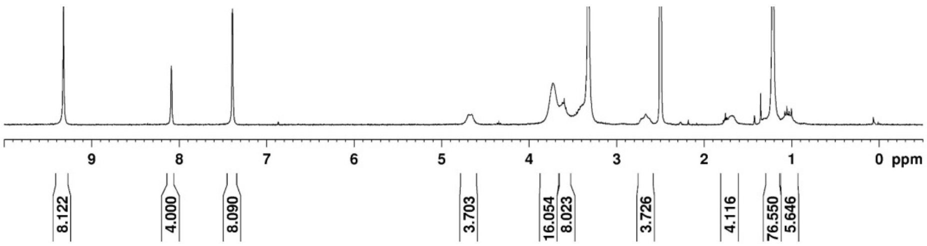

^{1}H-NMR of dendrimer **2** in DMSO-D6

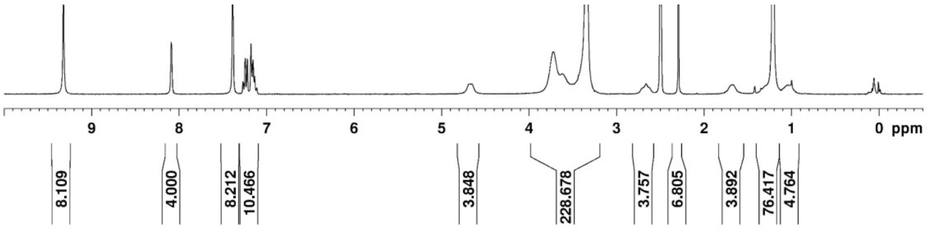

^{1}H-NMR of dendrimer **2** after adsorbing toluene in DMSO-D6

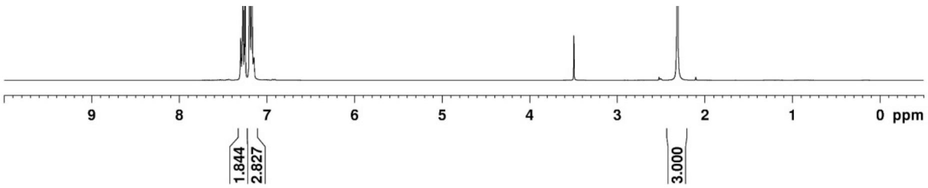

^{1}H-NMR of toluene in DMSO-D6

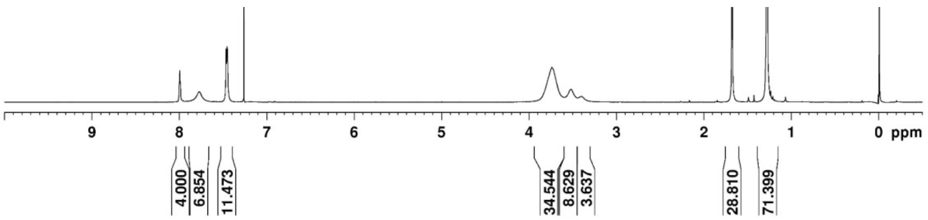

^{1}H-NMR of dendrimer **3** in $CDCl_3$

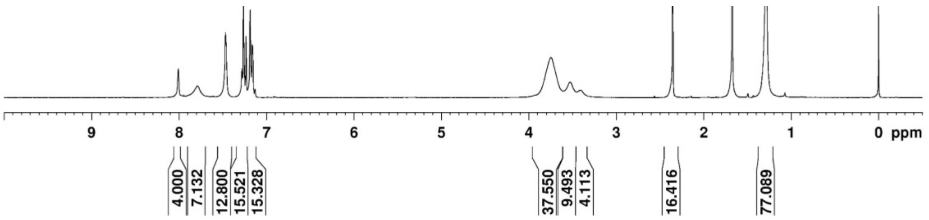

^{1}H-NMR of dendrimer **3** after adsorbing toluene in $CDCl_3$

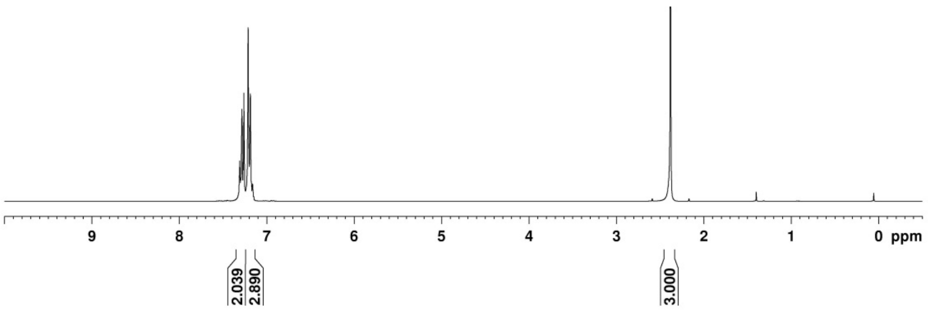

^{1}H-NMR of toluene in $CDCl_3$

(D) Adsorbing Hexane:

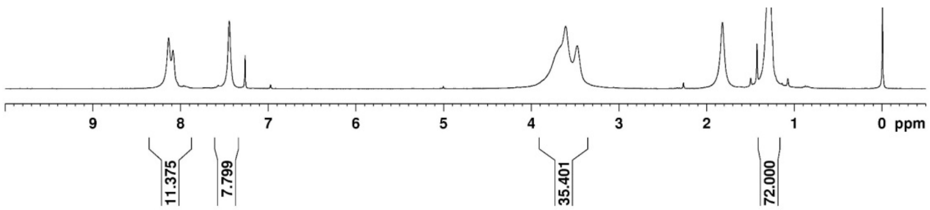

^{1}H-NMR of dendrimer **1** CDCl$_3$

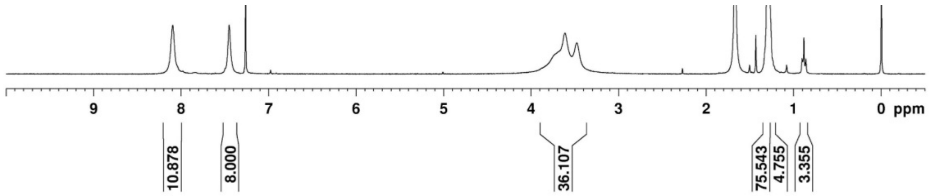

^{1}H-NMR of dendrimer **1** after adsorbing hexane in CDCl$_3$

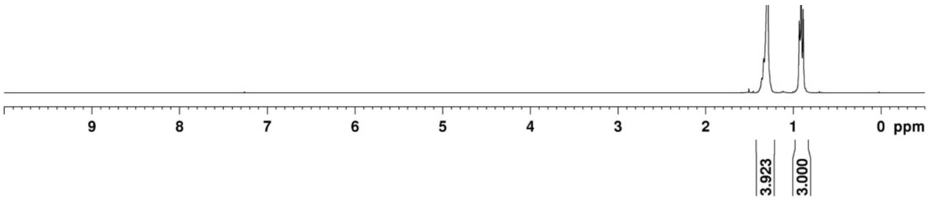

^{1}H-NMR of hexane in CDCl$_3$

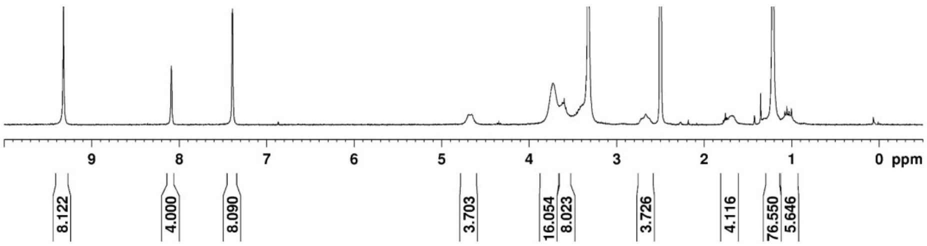

¹H-NMR of dendrimer **2** in DMSO-D6

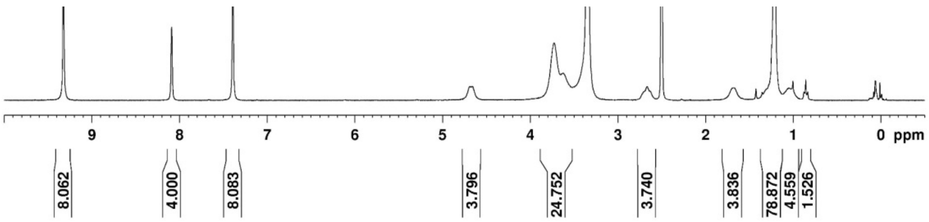

¹H-NMR of dendrimer **2** after adsorbing hexane in DMSO-D6

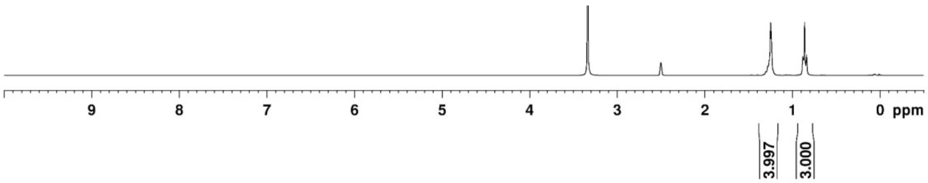

¹H-NMR of hexane in DMSO-D6

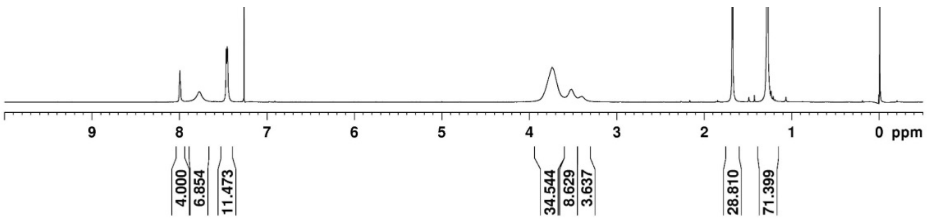

^{1}H-NMR of dendrimer **3** in $CDCL_3$

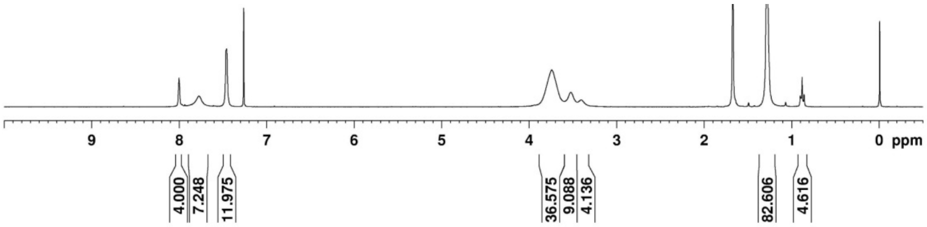

^{1}H-NMR of dendrimer **3** after adsorbing hexane in $CDCL_3$

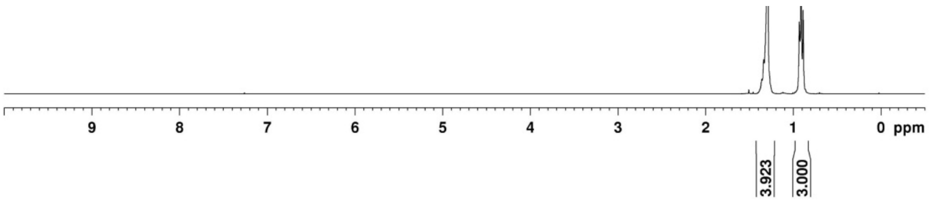

^{1}H-NMR of hexane in $CDCL_3$

Scheme S1. Estimation of isosteric heats of gas adsorption.

A virial-type equation **1** comprising the temperature independent parameters *ai* and *bi* was employed to calculate the enthalpies of adsorption for CO_2 (at 273 and 298 K).

$$\ln P = \ln N + 1/T \sum_{i=0}^{m} a_i N^i + \sum_{i=0}^{n} b_i N^i \qquad (1)$$

P: pressure, *N*: the amount adsorbed (or uptake), *T*: temperature, a_i and b_i: virial coefficients, and *m*, *n*: the number of coefficients required to adequately describe the isotherms (*m* and *n* were gradually increased until the contribution of the extra added *a* and *b* coefficients was deemed statistically insignificant towards the overall fitting, and the average value of the squared deviations from the experimental values was minimized). The values of the virial coefficients (a_0 to a_m) were then used to calculate the enthalpies heats of adsorption using the following expression.

$$Q_{st} = -R \sum_{i=0}^{m} a_i N^i \qquad (2)$$

Qst is the coverage-dependent isosteric heat of adsorption and *R* is the universal gas constant. The heat of CO_2 sorption for **2** and **3** in this manuscript is determined by using the excess sorption data in the pressure range from 0◎1 atm (273 and 298 K), which is fitted by the virial-equation very well.

Figure S3. The N_2 sorption isotherms of dendrimers **2** and **3** at 77K.

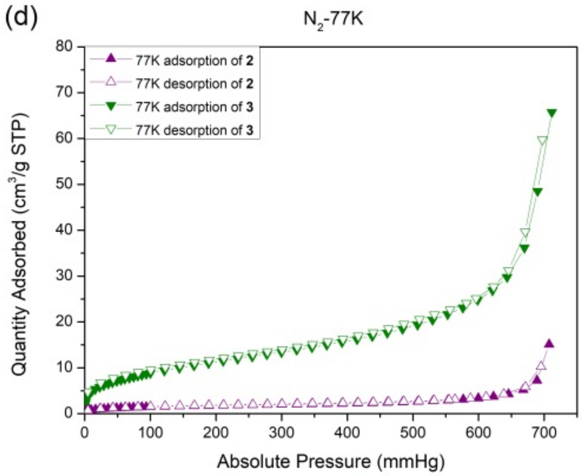

Figure S4. The pore size distribution of dendrimer **3** under nitrogen.

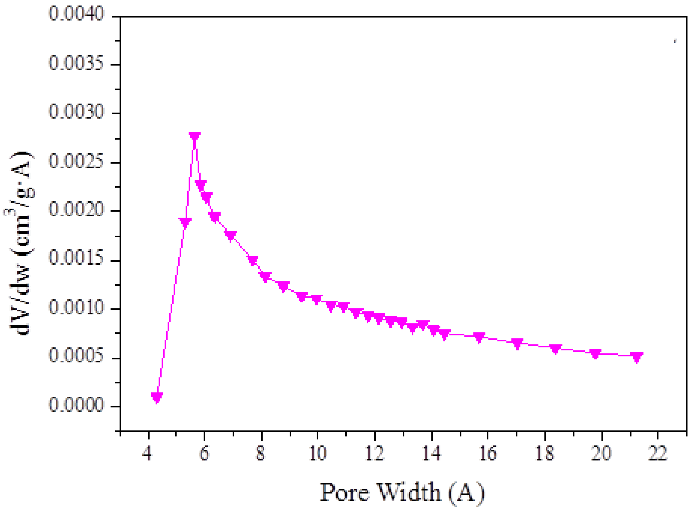

As the adsorption of nitrogen by dendrimer **2** is negligible, the pore size distribution is estimated by the adsorption of nitrogen by dendrimer **3**. There are two major pore sizes for **3**; the first one is ~5.8 Å and the second one is ~7.5 Å. The percentage of the first one is greater than the second one. The size of pyridine is less than those of nitrobenzene and toluene and this may explain that the adsorption of pyridine is greater than nitrobenzene and toluene by dendrimer **3**.

Figure S5. The ^{1}H-NMR and ^{13}C-NMR spectra of dendrimers **2** and **3.**

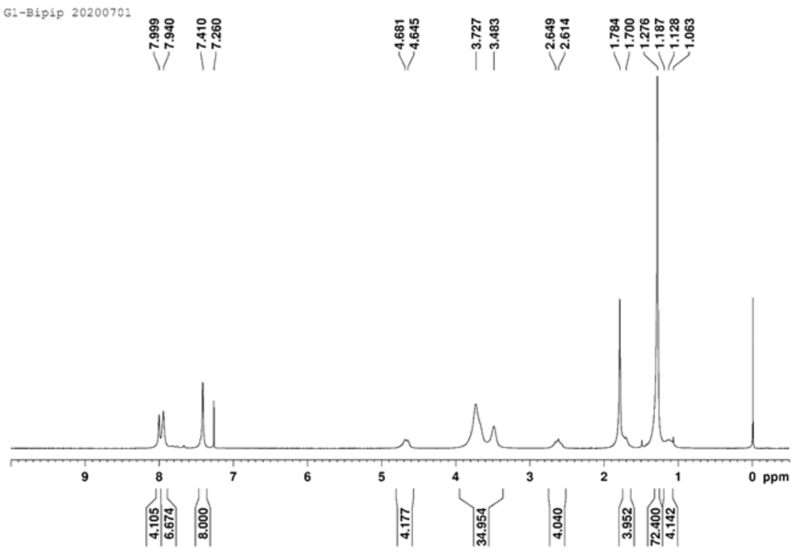

^{1}H-NMR spectrum of **2**

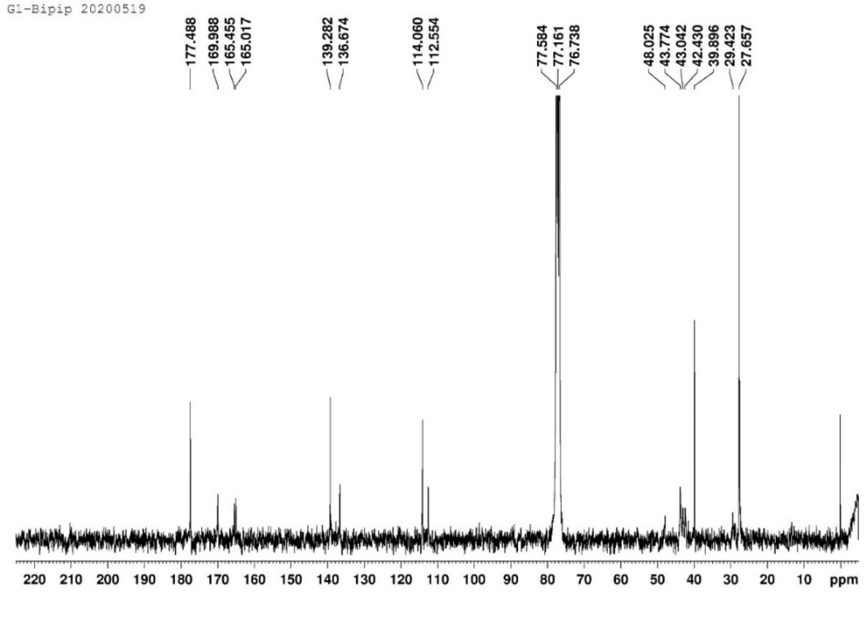

^{13}C-NMR spectrum of **2**

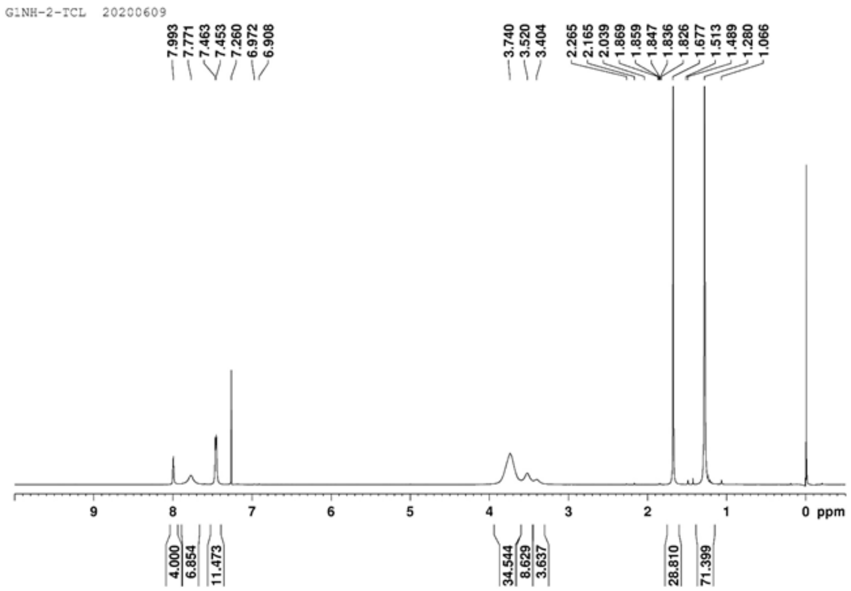

^{1}H^{1}H-NMR spectrum of **3**

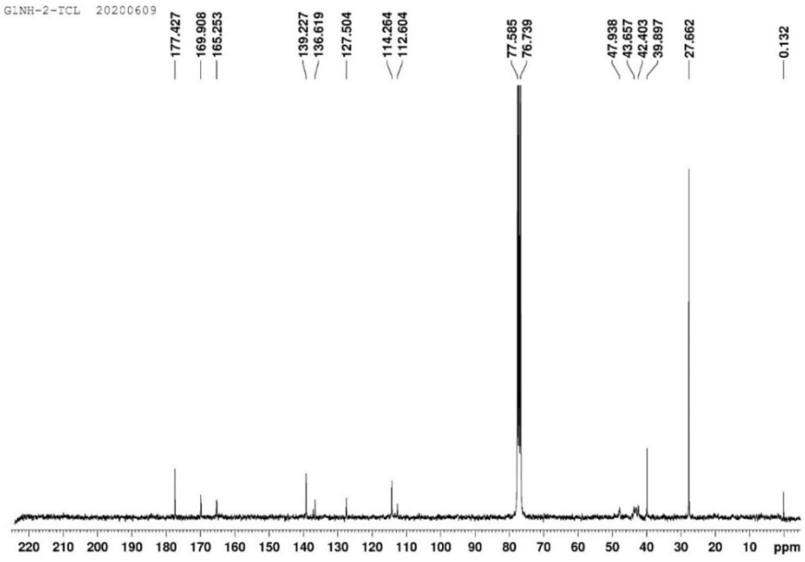

^{13}C-NMR spectrum of **3**
